# The geomicrobiology of CO_2_ geosequestration: a focused review on prokaryotic community responses to field-scale CO_2_ injection

**DOI:** 10.3389/fmicb.2015.00263

**Published:** 2015-04-09

**Authors:** Andre Mu, John W. Moreau

**Affiliations:** ^1^Moreau Lab, School of Earth Sciences, Faculty of Science, University of MelbourneMelbourne, VIC, Australia; ^2^Department of Microbiology and Immunology, Peter Doherty Institute for Infection and Immunity, University of MelbourneMelbourne, VIC, Australia

**Keywords:** CO_2_ geosequestration, deep subsurface, microbial response, CODH, systems biology, network analysis, methanogenesis, sulfur cycling

## Abstract

Our primary research paper (Mu et al., 2014) demonstrated selective changes to a deep subsurface prokaryotic community as a result of CO_2_ stress. Analyzing geochemical and microbial 16S rRNA gene profiles, we evaluated how *in situ* prokaryotic communities responded to increased CO_2_ and the presence of trace organic compounds, and related temporal shifts in phylogeny to changes in metabolic potential. In this focused review, we extend upon our previous discussion to present analysis of taxonomic unit co-occurrence profiles from the same field experiment, to attempt to describe dynamic community behavior within the deep subsurface. Understanding the physiology of the subsurface microbial biosphere, including how key functional groups integrate into the community, will be critical to determining the fate of injected CO_2_. For example, community-wide network analyses may provide insights to whether microbes cooperatively produce biofilm biomass, and/or biomineralize the CO_2_, and hence, induce changes to formation porosity or changes in electron flow. Furthermore, we discuss potential impacts to the feasibility of subsurface CO_2_ storage of selectively enriching for particular metabolic functions (e.g., methanogenesis) as a result of CO_2_ injection.

## Introduction

The carbon capture and storage (CCS) technology known as “geosequestration,” or injection of large volumes of supercritical CO_2_ (scCO_2_) into deep aquifers, has the potential to impact subsurface microbial community dynamics. Such impacts include changes to microbially-mediated terminal electron accepting processes that may, in turn, affect the geochemistry and mineralogy of the CO_2_ storage aquifer. Subsequent mineral precipitation or dissolution reactions, for example, could result in a decrease or increase in porosity that leads to redistribution of injected scCO_2_ (Gadd, 2010) or undesirable changes in groundwater chemistry. Other impacts may include methanogenesis as a result of locally increased dissolved CO_2_ levels under lowered pH conditions (Sato et al., 2013), or the inhibition of enzymatic carbon monoxide (CO) oxidation with broad consequences for microbial community carbon utilization and electron flow (Ragsdale, 2004; Techtmann et al., 2009). Accumulation of excess CO may increase the activity of acetotrophic methanogens, as CO is a metabolic intermediate and its concentration is known to be inversely related to that of methane in anaerobic digesters (Krzycki and Zeikus, 1984; Hickey and Switzenbaum, 1990). In fact, uncertainty currently exists around whether deep subsurface microbial responses to scCO_2_ injection will result in positive or negative effects on geosequestration. As an example, **biofilm** formation has been observed under scCO_2_ in laboratory bioreactors (e.g., Mitchell et al., 2009), and some researchers have proposed that this process may be stimulated in CO_2_ storage aquifers to help “lock in” scCO_2_ and prevent its migration as a buoyant plume. In contrast, other studies have observed that scCO_2_ injection led to the growth of methanogens (Morozova et al., 2011) and therefore raise the question of whether geosequestration could result in enhanced methane generation and potential leakage. Clearly, understanding the changes in microbial community composition and dynamics throughout geosequestration experiments, especially those conducted *in situ* under quasi-realistic conditions or in real CO_2_ storage aquifers, will provide useful insights for understanding the fate of scCO_2_.

KEY CONCEPT 1 **Biofilm**

Microbial biofilms are a community of mixed or single-isolate microbes encapsulated in a self-produced extracellular biopolymer matrix. Biofilms exhibit greater tolerance to CO_2_ stress, and have the potential to alter biogeochemical processes and reduce reservoir porosity.

Previous efforts to study microbial responses to scCO_2_ exposure have focused on characterizing the subsurface microbial community at the individual taxonomic level (Morozova et al., 2011; Bordenave et al., 2012; Lavalleur and Colwell, 2013). However, the resolution of these studies make apparent the need for **metagenomic** and **systems biology** -based analyses, such as functional gene and co-occurrence profiling, with a focus on elucidating potential **syntrophic** associations of importance to CO_2_ storage. We need to understand the microbial biosphere beyond simple biodiversity characterizations because unrelated lineages have been shown to converge in functional similarity or complementarity where lineage-environment associations are stable (Chaffron et al., 2010; Gadd, 2010). For example, different average genome sizes suggesting the presence of multiple taxa have been observed in redox selective environments (Raes et al., 2007; Angly et al., 2009), and dissimilatory Fe(III) reduction might be a common metabolic feature in deep subsurface petroleum reservoirs that are host to a range of anaerobic thermophiles and hyperthermophiles (Slobodkin et al., 1999). Another example might include observations of close spatial association between sulfate-reducing and sulfide-oxidizing bacteria or archaea in anoxic or acidic environments (Bond et al., 2000; Loy et al., 2004; Moreau et al., 2010). Furthermore, increasing consideration should be placed on incorporating the potential impacts derived from **operational-dependent engineering**, such as the emplacement of injection and sampling wells, into the analysis pipeline, as these can influence subsurface microbial community composition and function (Morozova et al., 2011; Bordenave et al., 2012; Lavalleur and Colwell, 2013; Mu et al., 2014). The same principles of systems biology apply to the study of many other subsurface environments, such as hydrocarbon rich reservoirs (Dojka et al., 1998; Golby et al., 2011; Joshi et al., 2014) and radioactive waste storage sites (Nazina et al., 2004; Chi Fru and Athar, 2008).

KEY CONCEPT 2 **Metagenomic**

Metagenomics involves the bioinformatic analysis of high-throughput sequencing data of whole community genomic material. Determining the functional gene profile will provide insights to potential biogeochemical processes that underpin microbial community dynamics.

KEY CONCEPT 3 **Systems biology**

Systems biology (systeomics) takes a holistic view of the biological system and assesses the community at a higher-order level. Analyses typically include computationally intensive co-occurrence profiling, and microbe-metabolism network associations.

KEY CONCEPT 4 **Syntrophy**

Also known as microbial cross-feeding, syntrophy describes the process by which one species feeds off the metabolic products of another species, thus potentially forming dependent relationships between phylogenetically distantly related microorganisms.

KEY CONCEPT 5 **Operational-dependent engineering**

The engineering required for CO_2_ geosequestration includes, for example, injection and sampling wells, which play an integral part of the project and may influence the geochemical milieu. Residual organic compounds from the drilling fluid used during emplacement of injection wells can influence microbial community composition and function.

Current literature is largely based on *in vitro* experiments that utilize representative geologic material to simulate CO_2_ geosequestration, and results indicate that single-isolate microbial biofilms exhibit greater tolerance to scCO_2_ stress compared to the planktonic phase of growth (Mitchell et al., 2008). Analysis of the *in vitro* community structure also showed the subsequent predominance of different taxonomic groups following exposure to CO_2_ stress (Mitchell et al., 2009). While it is essential to conduct *in vitro* studies, it is also important to conduct *in situ* field-scale experiments to (1) validate results obtained from lab-scale studies and (2) provide biological relevance in the context of real geology. To this extent, a field-based scCO_2_ sequestration experiment was conducted in the 1.4 km-deep Paaratte Formation of the Otway Basin, Australia (Paterson et al., 2013). One hundred and fifty tons of mixed scCO_2_ and groundwater was injected into the sandstone Paaratte aquifer over 4 days. Our primary research (Mu et al., 2014) detected changes in microbial community structure prior to scCO_2_ injection, which revealed a general shift from *Firmicutes* to *Proteobacteria* concurrent with the disappearance of polyethylene glycols (PEGs) that were interpreted as residual from drilling fluid used during the emplacement of the CO_2_ injection well. Furthermore, the persistence of *Carboxydocella*, *Comamonadaceae*, and *Sphingomonadaceae* after scCO_2_ injection suggested that these groups could adapt to the changes in groundwater chemistry resulting from the CO_2_ geosequestration experiment, including decreases in pH and temperature of 2.6 log units and 5.8°C, respectively, and an increase in CO_2_ concentration from 148 parts per million (ppm) to 1410 ppm. However, issues of activity (mRNA), metabolic function (transcriptome, proteome) and syntrophic interactions (network associations) of the subsurface biosphere still remain. Considering many environmental microbes are uncultivable in the laboratory (Rappé and Giovannoni, 2003; Schloss and Handelsman, 2004), scientists will need to employ multiple omics-based measurements (e.g., metagenomics, transcriptomics, etc.) and bioinformatic analyses (e.g., taxonomic co-occurrence profiling) coupled to geochemical measurements, to understand microbial physiology and the dynamic cycling of nutrients. Furthermore, to broaden our analysis, inductively coupled plasma mass spectrometry (ICP-MS) was performed to determine potential toxicity effects of CO_2_-induced mobilization of trace metals from the sediments into the water phase. With the exception of Fe, there were no discernable changes to the concentration of dissolved trace metals (including Ca, K, Mg, Al, Ba, Cu, Mn, Sr, and Zn) in Paaratte groundwater within the respective pre- and post-CO_2_ injection phases. Therefore, the observed changes to microbial community structure are unlikely to be attributable to potential metal toxicity. However, it is acknowledged that our results may not be universal for the reasons of different redox states and sediment types at different CO_2_-receiving reservoirs (Ardelan et al., 2009) where lowered pH conditions may facilitate the mobilization of trace elements. Therefore, this review aims to address some of the key issues, and to highlight the key gaps, in our understanding of environmental microbial responses to CO_2_ stress, and also present new analysis of microbial networks during a field-based scCO_2_ sequestration experiment.

## Materials and methods

### CO2CRC Otway Stage 2B

The Cooperative Research Centre for Greenhouse Gas Technologies (CO2CRC) Otway Stage 2B field experiment was conducted to ascertain the residual CO_2_ storage capacity of a sandstone aquifer, 1400 m true vertical depth sub-sea (TVDSS) in the Paaratte Formation (Otway Basin, Southeastern Australia), to assess geosequestration as a means to mitigate atmospheric CO_2_ pollution (Paterson et al., 2013). A summary of the scCO_2_ injection experiment is provided in Table 1.

**Table 1:**
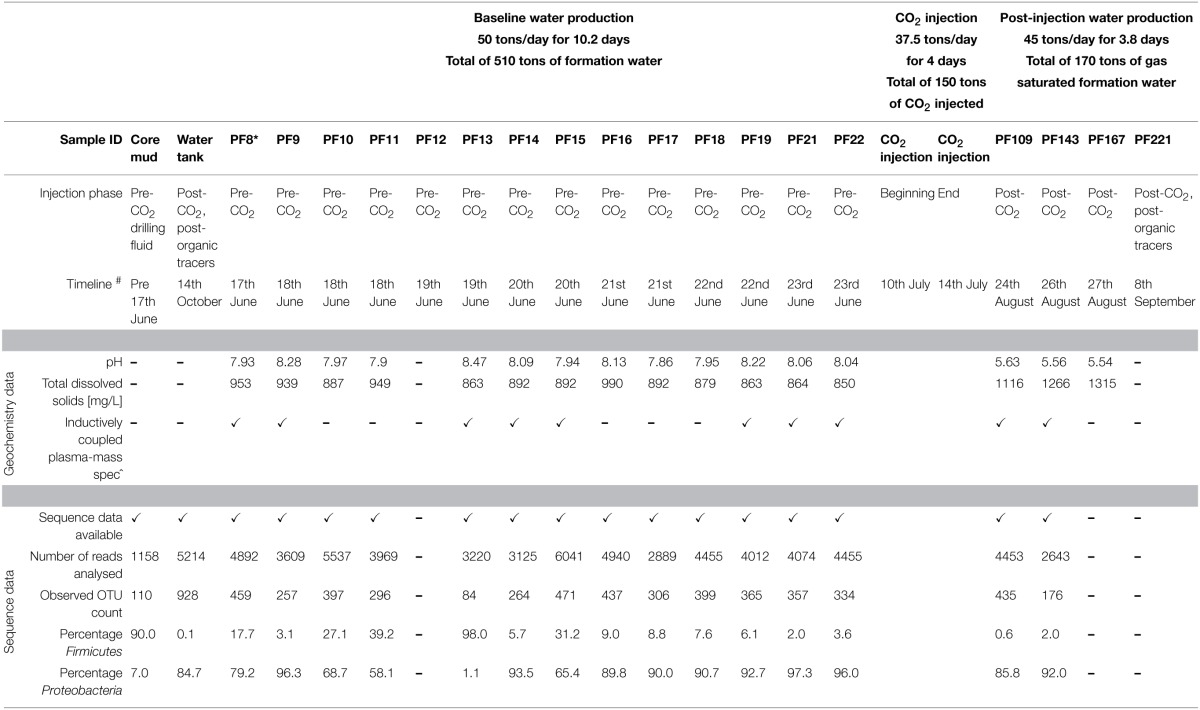
**Summary of baseline geochemical and bioinformatic analyses**.

^#^The temporal relationship, as indicated by the day and month, of each sample occurs over the year 2011.

-Data are unavailable due to dedicated sampling time points required by multi-disciplinary collaborative experiments.

*PF– – –; where PF represents “Paaratte Formation,” and – – – represents the number of U-tube sample since time origin.

^ All measurements were stable with the exception of dissolved Fe which decreased prior to CO_2_ injection and increased post-CO_2_injection.

Pristine water samples held under *in situ* conditions were obtained via a novel **U-tube** sampling system (see Section *In Situ Sampling of the Deep Subsurface Biosphere* for further information on the system) over the course of the scCO_2_ injection (150 tons) event for baseline geochemical analyses and genomic DNA extraction. A total of 79 U-tube water samples were collected. Whole community 16S rRNA gene profile analysis is described in detail in Mu et al. (2014). Briefly, biomass was concentrated, as 50 ml aliquots, on to 0.22 μm nylon filter membranes (Merk Millipore) using vacuum filtration, and processed for gDNA extraction using the MoBio Powersoil DNA extraction kit. Whole community gDNA was extracted onsite within 12 h of sampling. However, given the logistics of the sampling schedule post-scCO_2_ injection, filter membranes with concentrated biomass for nucleic acid extraction were stored on site in RNAprotect Bacteria Reagent (QIAGEN) at −20°C until processing could be performed in the laboratory. Whole community gDNA were amplified with native universal small subunit 803 forward and universal SSU1392w reverse (5′- ACG GGC GGT GWG TRC -3′) primers using High-fidelity OneTaq DNA polymerase mastermix (New England Biolabs). SSU803F primer is a combination of 803Fa 5′- TTA GAT ACC CTG GTA GTC -3′; 803Fb 5′- TTA GAT ACC CSG GTA GTC -3′; 803Fc 5′- TTA GAT ACC CYH GTA GTC -3′; 803Fd 5′- TTA GAG ACC CYG GTA GTC-3′; in a ratio of 2:1:1:1 for 803Fa:b:c:d. Amplicon sequencing was performed by the Australian Centre for Ecogenomics (ACE; University of Queensland, Australia) using barcoded 454 pyrosequencing (Roche). The QIIME bioinformatics pipeline was employed to analyse sequence data, assign taxonomy, and to determine phylogenetic distributions of each microbial community (Caporaso, 2010). Sequence data were clustered into operational taxonomic units at 97% pairwise identity using the UCLUST (Edgar, 2010) seed-based algorithm. A representative sequence from each OTU was aligned using the PyNAST tool (Caporaso et al., 2010) and queried against the Ribosomal Database Project (Wang et al., 2007) for taxonomy assignment. Results from the taxonomic classification using the Ribosomal Database Project classifier (Mu et al., 2014) through the QIIME tool (Caporaso, 2010) was analyzed to compute statistical dependence of each of the microbial orders, pre- and post-CO_2_ injection. The *otu.association* function from the Mothur software (version 1.27; Schloss et al., 2009) was used to calculate Spearman's rank correlation values of the taxonomic units based on their relative abundance percentages across all samples. Visualization of the corresponding networks with a coefficient cutoff value of 0.5, and one standard deviation away from the mean (i.e., 0.84), were aided by Cytoscape version 2.8.3 (Cline et al., 2007; Saito et al., 2012). The nodes represent microbial orders, while an edge indicates associations between connecting nodes. The *degree sorted circle* layout was imposed on the network to indicate a decreasing degree of associations between nodes (i.e., the number of nodes one OTU associates with) proceeding in a counter clockwise direction with the point of origin at the 180° position. Subnetworks within a community are denoted with alphabetical characters. The correlation test used in this study is the same as that found in the Phoenix 2 software for 16S rRNA gene analyses (Soh et al., 2013).

KEY CONCEPT 6 **U-tube**

A hydraulically sealed sampling system used to collect deep subsurface water and gas samples under *in situ* conditions. *In situ* water samples are collected and stored in high-pressure stainless steel cylinders (Swagelok) for downstream microbial and geochemical analyses.

## Results

### Community network analyses

**Figure 1:**
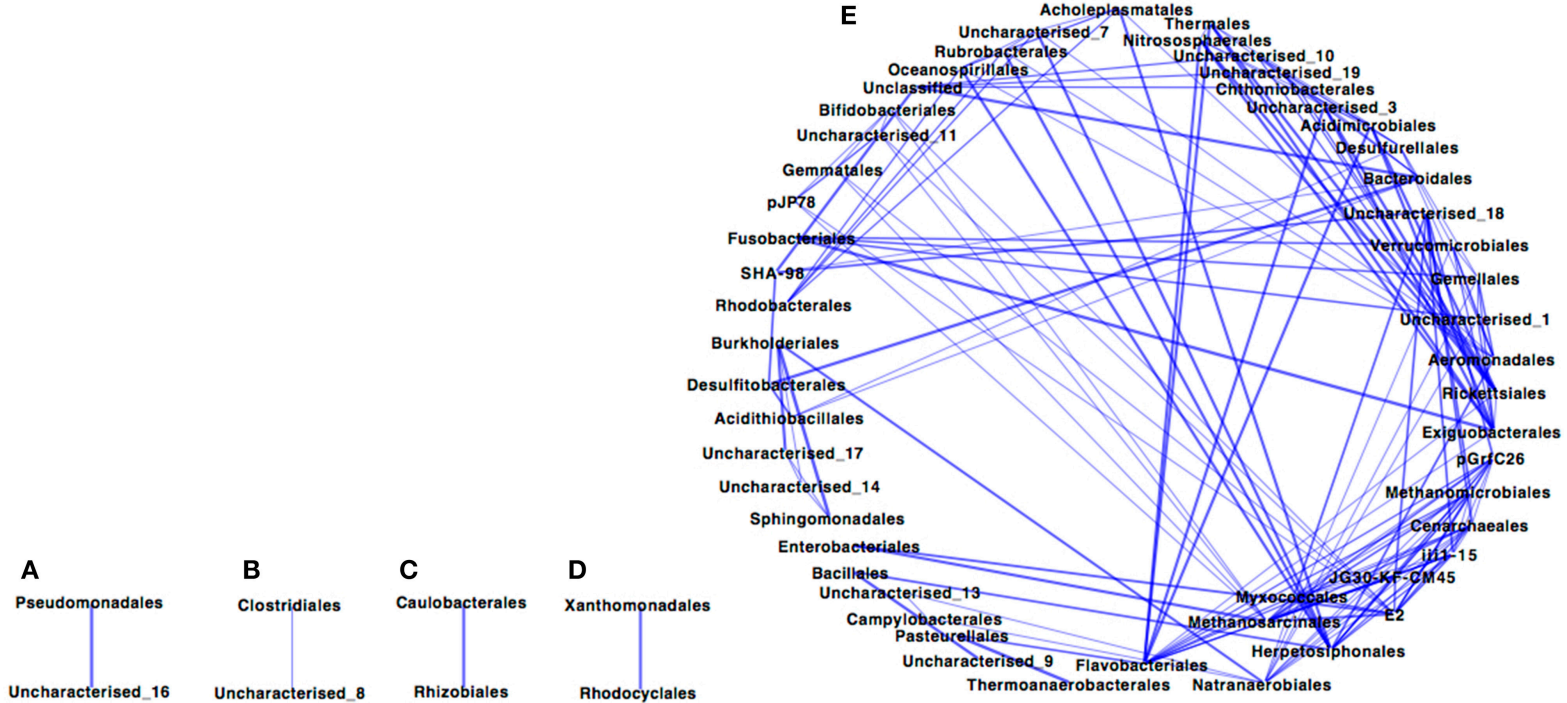
**Co-occurrence profile of the pre-CO_2_ injection Paaratte Formation microbial community with a Spearman's rank correlation of ≥0.5 (*p* < 0.025)**. Results from the taxonomic classification using the Ribosomal Database Project classifier through the QIIME tool was analyzed to compute statistical dependence of each of the microbial orders pre-CO_2_ injection. Spearman's Rank correlation values were calculated based on the relative abundance percentages of all taxonomic units across all samples using the *otu*.*association* function from the Mothur software (version 1.27). Networks were visualized using Cytoscape version 2.8.3. The nodes represent microbial orders, while an edge indicates an association between connecting nodes. The *degree sorted circle* layout was imposed on the network to indicate a decreasing degree of association between nodes proceeding in a counter clockwise direction (point of origin at the 180° position). That is to say the operational taxonomic unit at the 180° position associates with more OTUs than the others. Sub-networks are denoted with alphabetic characters.

The Paaratte Formation microbial community separated into five sub-networks (Spearman's rank correlation ≥ 0.5; *p* < 0.025) for samples obtained during the pre-CO_2_ injection phase (Figure 1). Sub-networks A, B, C, and D consisted of two taxonomical orders each, while, Sub-network E contained 52 orders. *Pseudomonadales*/*Pseudomonas* (Order/Genus), the predominant *Proteobacteria*, associated with an uncharacterized taxon in Sub-network A. Similarly, *Clostridiales/Carboxydocella* (Order/Genus), an anaerobic thermophile, associated with an uncharacterized taxon in Sub-network B. *Sphingomonadales/Sphingomonadaceae* (Order/Family) and *Burkholderiales/Comamonadaceae* (Order/Family), which were shown to proliferate in relative abundance during the post-CO_2_ injection phase (Mu et al., 2014), are illustrated in Sub-network E to have an underlying association with 87% of the microbial community at the order level of classification. However, the degree of association for *Sphingomonadaceae* and *Burkholderiales* was relatively weak as the orders were positioned further away from point origin. Furthermore, sulfur cycling-associated taxa, i.e., *Acidithiobacillales* and *Desulfitobacterales* (Table 2) were integrated into Sub-network E (Figure 1). The microbial community as visualized at a Spearman's rank correlation ≥ 0.84 (*p* < 0.005) illustrated a strong interaction between *Methanomicrobiales* and *Myxococcales* (Table 2, and Figure 2 sub-network G). The most abundant OTUs, *Pseudomonadales*/*Pseudomonas* and *Clostridiales/Carboxydocella*, were absent from the entire network at a Spearman's rank correlation cutoff ≥ 0.84 (*p* < 0.005; Figure 2).

**Table 2:** **Spearman's rank correlation coefficient and corresponding *p*-values for associating operational taxonomic units**.

**Operational taxonomic unit A^#^**	**Positive correlation coefficient**	**Operational taxonomic unit B**	***p*-value**
*Burkholderiales*	0.619804	*Verrucomicrobiales*	0.025
*Burkholderiales*	0.606026	*Caulobacterales*	0.025
*Burkholderiales*	0.591434	*Uncharacterised_13*	0.025
*Burkholderiales*	0.642945	*Uncharacterised_17*	0.025
*Clostridiales*	0.790928	*Uncharacterised_8*	0.025
*Desulfitobacterales*	0.684653	*Acidithiobacillales*	0.025
*Myxococcales*	1	*Methanomicrobiales*	0.005
*Pseudomonadales*	0.651935	*Uncharacterised_16*	0.025
*Sphingomonadales*	0.766587	*Alteromonadales*	0.025
*Sphingomonadales*	0.525105	*BD7-3*	0.025
*Sphingomonadales*	0.620098	*Burkholderiales*	0.025
*Sphingomonadales*	0.626152	*Rhizobiales*	0.025

# *Operational taxonomic unit A correlates with operational taxonomic unit B at a coefficient value indicated in the second column. P-values support the significance of the correlation values based on number of samples anayzed*.

**Figure 2:**
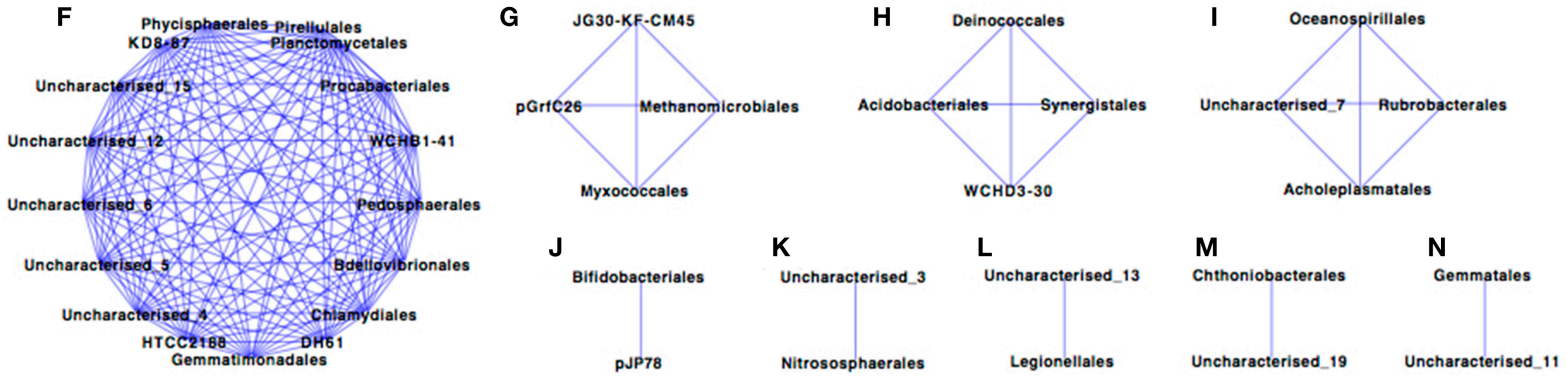
**Co-occurrence profiling of the pre- and post-CO_2_ injection Paaratte Formation microbial community with a Spearman's rank correlation of ≥0.84**. Results from the taxonomic classification using the Ribosomal Database Project classifier through the QIIME tool was analyzed to compute statistical dependence of each of the microbial orders pre- and post-CO_2_ injection. Spearman's Rank correlation values were calculated based on the relative abundance percentages of all taxonomic units across all samples using the *otu*.*association* function from the Mothur software (version 1.27). Networks were visualized using Cytoscape version 2.8.3. The nodes represent microbial orders, while an edge indicates an association between connecting nodes. The *degree sorted circle* layout was imposed on the network to indicate a decreasing degree of association between nodes proceeding in a counter clockwise direction (point of origin at the 180° position).

## Discussion

### Logistics of a carbon geosequestration project

Ongoing investigations are aimed at understanding the feasibility of capturing and sequestering industrial volumes of CO_2_ within the Earth's subsurface, in an attempt to reduce the impacts of anthropogenic climate change. Carbon capture and storage is steadily proving to be a viable solution in cutting global CO_2_ emissions (Benson and Surles, 2006; Gibbins and Chalmers, 2008). However, a number of factors need to be considered before injecting many kilotons of CO_2_ into the subsurface, including, for example, site selection (Bachu, 2000, 2002; Ennis-King et al., 2011), geochemical and biological monitoring & verification (Stalker et al., 2009; Réveillère and Rohmer, 2011; Schacht et al., 2011; Jenkins et al., 2012; Noble et al., 2012), engineering and sampling representative material for analysis (Freifeld et al., 2005; Kharaka et al., 2006; Freifeld, 2009), and the mechanism by which CO_2_ is sequestered (Li et al., 2006; Saadatpoor et al., 2009). The Australian CO2CRC Otway project provides insights into factors associated with field-scale experimental design (Sharma et al., 2009; Boreham et al., 2011; Underschultz et al., 2011; Haese et al., 2013) that may have implications for understanding subsurface biosphere responses to CO_2_ geosequestration. More details about each of these factors can be found in the original references, and several are discussed below.

#### Engineering derived influences on biodiversity

In the context of geosequestration, “engineering” includes controlling the flow rate of water sampling, the injection rate of gas-saturated water, and the use of drilling fluid during the emplacement of injection and sampling wells. Operational-dependent engineering can have indirect impacts on the characterization of the microbial community. For example, a high flow rate of water sampling may force attached bacterial cells (sessile population) to detach into pore waters for analysis (MacDonald et al., 1999). Whereas, low flow rates may only be sampling planktonic community members for analysis due to reduced shearing forces (MacDonald et al., 1999), and thereby under-represent the complete microbial biosphere. Surface-associated physiochemical properties could also influence the affinity of cell attachment as a function of hydrophobicity (Donlan, 2002; Mitchell et al., 2009). An understanding of site-specific hydrogeology, and collection of both fluid phase and core samples (Kolbel-Boelke et al., 1988; Godsy et al., 1992; Bekins et al., 1999), will help to evaluate microbial community responses to increased CO_2_ levels.

#### *In situ* sampling of the deep subsurface biosphere

Understanding the microbial biosphere of deep subsurface environments requires precise and “sterile” sampling of representative geologic material, which is often difficult to achieve because very few techniques allow for subsurface samples to be retained under *in situ* pressure and temperature conditions (e.g., Schlumberger's MDT syringe-like tool; and evacuated Kuster samplers, Kuster Company, Long Beach, Ca, USA; Freifeld et al., 2005; Kharaka et al., 2006). Therefore, to obtain geochemically pristine water samples held under *in situ* conditions, while not compromising the stability of injected CO_2_, a novel, hydraulically sealed “U-tube” sampling system (Freifeld et al., 2005; Freifeld, 2009) was employed in our primary research paper (Mu et al., 2014). The U-tube system produces formation water to the surface using directive flow of high-pressure nitrogen gas into specialized pressure cylinders independent of the injection line. Furthermore, engineering of the U-tube allows for the isolation, and storage of formation water under conditions that are characteristic of subsurface environments. This sampling mechanism is particularly important for deep, anoxic subsurface environments where even slight exposure to oxygen can be harmful to the sampled microorganisms (Fredrickson and Onstott, 1996). Therefore, by using the U-tube system the microbial community may be analyzed as close to its *in situ* physiological state, whether it is through culture-dependent and/or -independent assays.

#### Residual sequestration of CO_2_

Residual sequestration is characterized by the formation of plumes when free-phase CO_2_ migrates and becomes trapped by capillary pressure from the water in the pore spaces between the rocks. The mechanism thus prevents CO_2_ from flowing, and effectively sequesters the injected CO_2_ (Nghiem et al., 2009; Zhang et al., 2011; Shamshiri and Jafarpour, 2012). Residual trapping was the method of sequestration used in our primary research paper. The mechanics of residual trapping alludes to the possibility of creating microenvironments that may select for, and/or provide the conditions in which, different syntrophic microbial networks co-occur within the reservoir. Indeed, regions with trapped CO_2_ can affect the immediate environments geochemistry (e.g., pH, solubility), and in turn alter microbe-surface interactions. The concept of microenvironments is discussed further in the following sections.

It is probable that over geological time scales the buoyant CO_2_ plume may escape residual sequestration, migrate upwards and subsequently affect microbial community structure and dynamics. Therefore, recent studies (Oppermann et al., 2010; Morales and Holben, 2014) have investigated the impacts of CO_2_ leakage on surface soil communities. Similar to our original research (Mu et al., 2014), Morales and Holben (2014) reported a community structure predominated by *Proteobacteria* and low abundances of *Firmicutes* after exposure to CO_2_. Furthermore, they measured a statistically significant decrease in species richness, albeit only 3%. Reduced species richness has implications in ecosystem functional potential whereby a loss of diversity can translate to a loss in ecological functions (Tilman, 1999; Loreau et al., 2001; Petchey and Gaston, 2006), thus, affecting the cycling of carbon and other energy sources.

### *In vitro* studies

It is difficult to ascertain whether subsurface microorganisms biomineralize and/or convert the injected CO_2_ into biomass. Therefore, *in vitro* bioreactor studies are conducted to elucidate the mechanisms behind biogeochemical processes that play key roles in determining the fate of injected CO_2_. Of particular interest is how microbial biofilms might impact community dynamics and the sequestration of CO_2_; it is known that microbial biofilms have the potential to tolerate short exposures to scCO_2_ stress, and reduce reservoir porosity (Mitchell et al., 2008). For example, Mitchell and colleagues hypothesized that the protective effects of extracellular polymeric substances—through offering mass transport resistance, and large surface areas—resulted in the resilience of *Bacillus mojavensis* biofilms after exposure to scCO_2_ stress (Mitchell et al., 2008). Using a high-pressure flow reactor (1290 psi, 32°C) and electron microscopy, they also showed that sandstone cores, initially inoculated with biofilm forming *Shewanella frigidimarina*, were later populated with viable, sandstone-native, biofilm-forming cells of *B. mojavensis* and *Citrobacter* sp., after scCO_2_ and nutrient starvation stress. Observation that the microbial community changes after CO_2_ stress indicates the importance in understanding which taxa are being selected for, and their physiology, in order to predict their responses to increased CO_2_ levels. However, many *in vitro* analyses have so far been restricted to phenotypic assessment, which falls short of the resolution required to predict the behavior of biofilms and planktonic populations. It is therefore crucial that we understand the molecular mechanisms (i.e., genomic, metagenomic, transcriptomic, metabolomic, and proteomic) behind biofilm formation, biofilm-induced calcium carbonate precipitation (Phillips et al., 2013), and general microbial tolerance to CO_2_ stress, in order to control and utilize microorganisms for remediation purposes, such as long-term sealing of fractures in subsurface storage reservoirs. A recent study analyzing the transcriptome of a model sulfate-reducing microorganism, *Desulfovibrio vulgaris*, identified that cells up-regulated the transcription of certain amino-acids related to osmotic stress responses, and genes associated with chemotaxis (e.g., flagella subunits), in response to elevated CO_2_ pressures (Wilkins et al., 2014). However, scientists are routinely tasked with collecting adequate amounts of quality nucleic acid, in the form of biomass, from the deep subsurface to conduct the aforementioned omics measurements; modifying the U-tube system to collect significantly larger volumes of subsurface waters may be one option. Nevertheless, the real value in resolving such technical issues associated with omics-based measurements will be reflected upon when the physiological roles (i.e., transcriptome, proteome) that microorganisms play in the dynamic cycling of biogeochemically relevant elements, particularly the physiology of prokaryotes that are uncultivable *in vitro*, are determined (Handelsman, 2004; Lasken, 2012).

### Field-scale studies

There are a number of factors that might contribute to apparent disconnects between data generated from laboratory-based experiments and those obtained from field-scale projects, such as, hydrogeology (e.g., flow rates), rates of CO_2_-induced mobilization of contaminants (including trace metals), CO_2_-induced pH changes, unknown native reservoir gas composition, and intrusion of groundwater from nearby aquifers (refer to Harvey et al., 2013 for a detailed review on the geochemical implications of geosequestration). Such parameters have implications on evaluating the feasibility and potential of certain geological formations as CO_2_ storage sites for the reason that they can significantly influence the physiological state of the microbial community, and in turn alter the cycling of biogeochemically-relevant elements (Chapelle, 2000; Allen et al., 2007). For example, certain net redox reactions may be strongly acid consuming and thus highly sensitive to pH shifts (e.g., manganese and iron reduction), while others (e.g., sulfate reduction and methanogenesis) are less sensitive to CO_2_ induced pH changes (Bethke et al., 2011). Therefore, to better account for the inherent difficulties in detailing the intricate dynamics of the subsurface microbial biosphere, advances in sampling systems, such as the U-tube system (Freifeld et al., 2005; Freifeld, 2009) which provides fluid and gas samples retained under *in situ* conditions, can perhaps yield the necessary insights to design “geomicrobially representative” synthetic communities for *in vitro* culturing assays. Furthermore, there needs to be an ongoing process whereby results from laboratory experiments are analyzed and interpreted alongside data collected from *in situ* field-scale projects. More specifically, the experimental design of laboratory-based studies should incorporate site-specific geology and microbiology.

Valid concerns have been raised about whether studied subsurface communities are representative, especially when aerobic and facultative anaerobic taxa are detected in bioinformatic analyses of what should presumably be anaerobic environments (Balkwill, 1989; Sinclair and Ghiorse, 1989; Chandler et al., 1997; Biddle et al., 2008; An et al., 2013; Kimes et al., 2013). An et al. (2013) showed that hydrocarbon resource environments (HRE) commonly harbor an unexpected population of aerobic taxa and genes. The authors reasoned that some HREs might have available oxygen through infiltrating precipitation-derived waters (Andriashek and Atkinson, 2007; An et al., 2013). Furthermore, oxygenated groundwaters could be more prevalent in the subsurface than what is presumed and hence create oxic microenvironments that could allow aerobic respiration to persist in predominantly reducing environments (Winograd and Robertson, 1982). Alternatively, we speculate that operational–dependent engineering, such as sampling wells, may seed aerobic communities (or facultative anaerobes) into the subsurface. Because these communities, including eukaryotes (Orsi et al., 2013; Rédou et al., 2014), may play a significant role in the cycling of trace elements, they need to be assessed as apart of the ecosystem (e.g., CO_2_ geosequestration, oil recovery etc.). That is to say, for example, CO_2_ geosequestration will not be complete without the emplacement, and utilization of injection and sampling wells. Therefore, we must include potential engineering-derived communities and site-specific metadata (e.g., geochemistry [dissolved oxygen], lithology, hydrogeology/groundwater recharge, spatial and temporal heterogeneities) in the analysis process.

In order to predict what might happen to injected CO_2_, and understand the microbial influences on geosequestration at a field-scale level, Bordenave et al. (2012) characterized the baseline microbial community of a salt cavern (Baker Hughes, Alberta, Canada), while Lavalleur and Colwell (2013) focused on a basalt formation (Wallula pilot Eastern Washington State, USA). Pyrosequencing analysis showed that the anaerobic salt cavern was predominated by a halophilic, and thermophilic community. Further analysis by Bordenave et al. (2012), indicated that homoacetogenic activity was present at high and low salt concentrations, while methanogenesis was only present at low salt concentrations in the presence of H_2_. Similarly, Lavalleur and Colwell (2013) characterized the pre-CO_2_ injection biosphere of a basalt formation and demonstrated, through pyrosequencing, that *Proteobacteria*, *Firmicutes* and *Actinobacteria* were the predominant taxa. Furthermore, the closest known relatives as determined by 16S rRNA gene sequence similarity suggested the presence of H_2_-oxidizers, methylotrophs, sulfate reducers, methanotrophs, and methanogens (Lavalleur and Colwell, 2013), and thus, implied that hydrogen and single carbon-compounds might play significant roles in sustaining the deep biosphere (Stevens and McKinley, 1995). A study by Morozova et al. (2011) characterizing the microbial community of a siltstone and sandstone environment using fluorescent *in situ* hybridization (bacteria, archaea, and SRB 16S rRNA probes) and 16S rRNA gene fingerprinting (single-strand conformation polymorphism, and denaturing gradient gel electrophoresis) showed that fermentative halophilic bacteria (*Haloanaerobium* sp., *Halobacteroidaceae*) and SRB (*Desulfohalobium* sp., *Desulfotomaculum* sp.) were the dominant members of the community. This study represented one of the first efforts to characterize the biosphere throughout a CO_2_ geosequestration project, but lacked the sensitivity of high throughput DNA sequencing. Combining high throughput sequencing and organic geochemistry analyses, our principle research paper evaluated how *in situ* bacterial populations responded to increased CO_2_ levels and the presence of residual organic compounds, and related the temporal shift in taxonomic grouping (i.e., *Firmicutes* to *Proteobacteria*) to a switch in metabolic potential (Fermentation of residual organic compounds to respiration; Mu et al., 2014). Furthermore, our results suggested the potential for enhanced scCO_2_ tolerance, including changes in response to the associated variables (e.g., pH, temperature, and salinity), by *Comamonadaceae* and *Sphingomonadaceae*.

High variability exists in microbial community structure for *all* sites analyzed to date for geosequestration and other subsurface environments. The observed diversity of functional groups within geochemically defined environments might obscure syntrophic behaviors that are not readily discernable through relative abundance analyses of taxonomy data alone. This observation supports the need to incorporate *systems biology* approaches to develop an understanding of the links that underpin the networks and interactions of subsurface microbial communities in response to anthropogenic change. A community-wide approach that incorporates the analysis of microbial co-occurrences might reveal characteristics such as microbial cross-feeding that links phylogenetically distant microbes in a syntrophic relationship. For example, the degradation of natural organic matter in the subsurface by fermenting microbes generates bicarbonate (HCO^−^_3_), hydrogen (H_2_), and simple organic compounds (e.g., CH_3_COO^−^), which in turn may be utilized by hydrogenotrophic and acetotrophic methanogens to produce methane (Demirel and Scherer, 2008), or by sulfate and iron reducers as substrates for anaerobic respiration (Nealson and Saffarini, 1994). Another example might include competitive exclusion, a phenomenon that gives functional groups of microbes with a higher potential on the thermodynamic redox ladder an advantage over groups lower on the ladder that compete for the same electron donor (Lovley and Phillips, 1987; Chapelle and Lovley, 1992; Hoehler et al., 1998; Heimann et al., 2010; Bethke et al., 2011). We need to understand community functional dynamics, rather than solely taxonomic diversity, in order to delineate community functions and understand how the microbial biosphere responds to perturbation events such as CO_2_ injection and/or trace metal contaminants.

### Co-occurrence profiling reveal insights into microbial community functions

Preliminary results from co-occurrence analyses indicate that the Paaratte Formation microbial community may have underlying similarities in biogeochemical pathways and function as an interactive consortium. An association of the majority of the community (Figure 1 Network E) suggests a high degree of interdependency between the taxonomic units. However, separation of the community into five networks during the pre-CO_2_ injection phase (Figure 1) alludes to the presence of different co-occurring system behaviors, including narrow and syntrophic associations. The following subsections provide a brief overview of two main metabolic functions frequently characterized at geosequestration sites.

#### Methanogenesis

A pertinent question in carbon geosequestration is, “Will the injected CO_2_ stimulate a community that is overrepresented by microorganisms that can convert CO_2_ to CH_4_?”. Previous studies have highlighted the presence of methanogens pre-perturbation in subsurface biospheres that are targeted for CO_2_ geosequestration (Bordenave et al., 2012; Lavalleur and Colwell, 2013). Furthermore, methane is shown to be in high concentrations in basaltic formations (1,270 m depth; Stevens et al., 1993; Stevens and McKinley, 1995), and any leakage from CO_2_ injection-induced events will also trigger the leakage of native reservoir gases (Harvey et al., 2013). Methane is a primary contributor to climate change and is more potent as a greenhouse gas when compared to CO_2_ (Lashof and Ahuja, 1990; Shindell et al., 2009). Therefore, the concern for CCS becomes a question of, “Are we solving one environmental problem (atmospheric CO_2_ pollution) at the expense of creating another (e.g., methane pollution)?”

**Figure 3:**
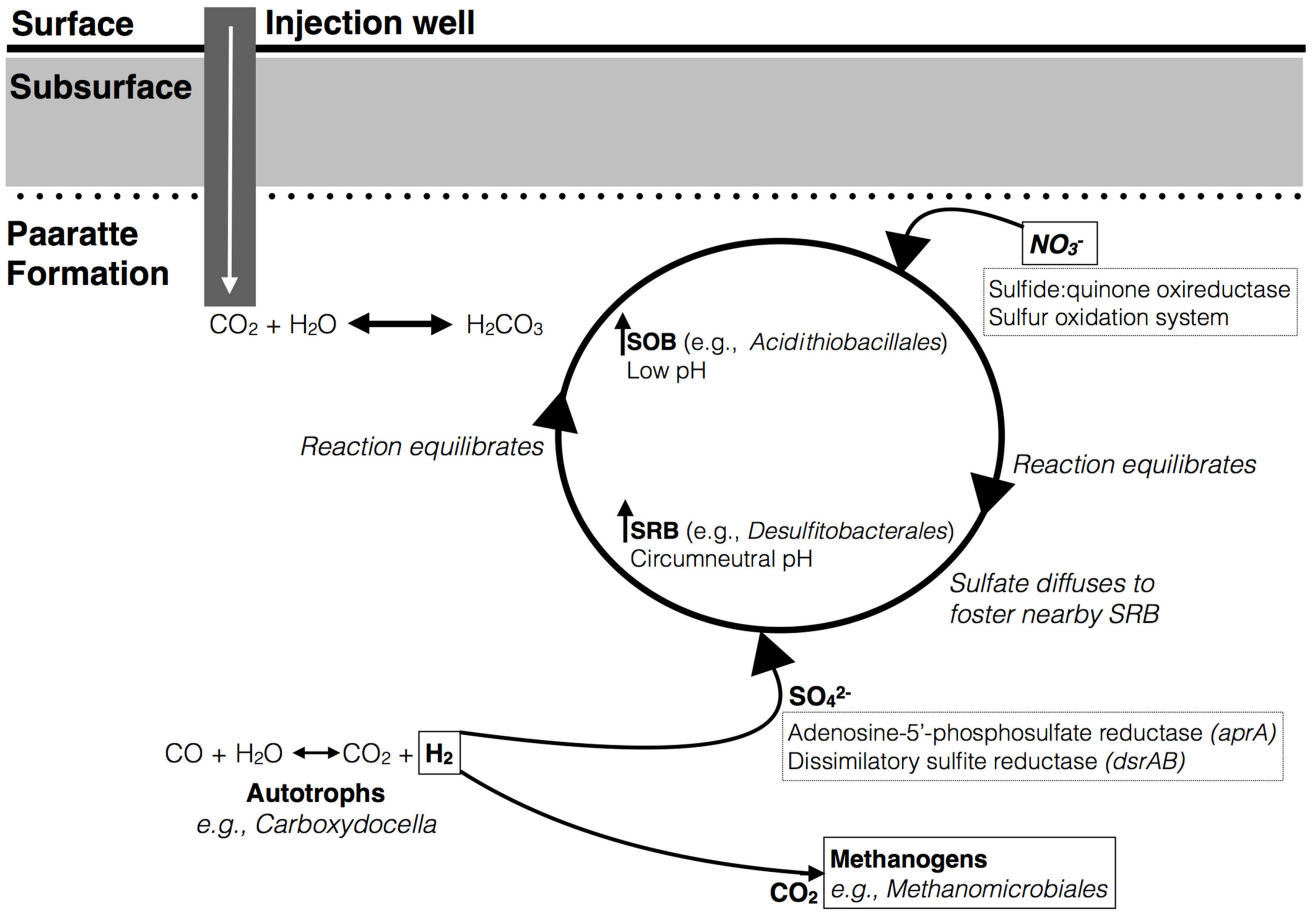
**A working model to explain the co-occurrence of sulfur-oxidizing and -reducing bacteria in network analyses**. A decrease in groundwater pH as a result of CO_2_ injection selects for growth of sulfide-oxidizing bacteria (SOB). The oxidation of sulfur may be coupled to nitrate as a terminal electron acceptor to proceed anaerobically. This reaction produces sulfate that can diffuse to allow for the activity of nearby sulfate-reducing bacteria (SRB) under circumneutral pH conditions. Autotrophs (e.g., carboxydotrophs) are a source of H_2_ for sulfate-reducers to metabolize the available SO^2−^_4_. The genes that encode for the potential enzymes responsible for oxidizing and reducing sulfur are respectively highlighted (in dashed boxes). Furthermore, it is illustrated that carboxydotrophs can also supply methanogens with CO_2_ and H_2_ required for methanogenesis.

Microbes are important terminal oxidizers during the anaerobic mineralization of organic matter to CO_2_ and CH_4_ in low sulfate environments. Methanogenesis occurs slowly in the presence of sulfate-reducing bacteria because sulfate reducers have a higher affinity for hydrogen and acetate, and there is a higher energy yield from sulfate reduction (Schonheit et al., 1982; Lovley and Klug, 1983). Therefore, SRB tend to outcompete methanogens. However, Morozova et al. (2011) demonstrated the temporary dominance of methanogenic archaea over SRB during CO_2_ storage in a saline aquifer (Ketzin, Germany). The observation by Morozova and colleagues highlights the question of potential stimulated methane production and subsequently, the potential for CO_2_ and methane co-leakage from a storage aquifer. Preliminary co-occurrence analyses of microbial taxa from the Paaratte Formation (Figure 3), however, demonstrate the association (>84% co-occurrence) between *Methanomicrobiales* and *Myxococcales* (Table 2; Figure 2). *Methanomicrobiales* is a CO_2_-reducing methanogen that uses H_2_ (or formate) as the reducing agent (Sakai et al., 2012). On the other-hand, *Myxococcales* is an organism that feeds solely on insoluble organic substances (Dawid, 2000; Zhou et al., 2014), and has been proposed to associate with methanogens through dependent predation on methanotrophs (Osaka et al., 2008). Therefore, co-occurrence of the two taxa suggests that some methane cycling may have occurred with a subsequent contribution to (recycled) biomass.

The results provide a basis for testing the hypothesis that a dependent relationship can form around methanogenesis during a CO_2_ geosequestration project. Further culture-based experiments should therefore include targeted analysis of **autotrophic** consumption of CO_2_ and methane production.

KEY CONCEPT 7 **Autotrophy**

Autotrophy describes the process by which microorganisms produce complex organic compounds from simple inorganic carbon molecules.

#### Cyclic metabolism of sulfur

Sulfur exists in multiple oxidation states with an eight-electron difference between the two end members, from sulfide to sulfate. Multiple microbial members are required to carry out the series of reaction pathways of sulfur oxidation intermediates (Vairavamurthy et al., 1993; Detmers et al., 2001; Friedrich et al., 2005). Preliminary analysis of the Paaratte Formation has suggested a co-occurrence of sulfide-oxidizing bacteria (SOB) *Acidithiobacillales*, and sulfur-reducing bacteria, *Desulfitobacterales* (Table 2). Associations between SOB and SRB in the environment have been described in the past (Elshahed et al., 2003). A working model is presented in Figure 3, which hypothesizes an association between the SRB and SOB within microenvironments created by the residually trapped CO_2_. We speculate that a geochemical link establishes between the sulfur cyclers within the reservoir whereby the decrease in pH as a result of CO_2_ injection selects for SOB (Harrison, 1984). This reaction may produce sulfate that can diffuse to foster the activity of nearby SRB under circumneutral pH conditions. Autotrophs (e.g., *Carboxydocella*) are potential sources of H_2_ for sulfate-reducers to metabolize the available SO^2−^_4_ (Techtmann et al., 2009). However, results from our original research suggest the potential inhibition of CO-oxidation by the thermophilic autotroph, *Carboxydocella*, as a result of the CO_2_ injection project. The inhibition of enzymatic CO-oxidation could therefore have broad implications for the cycling of carbon, including methanogenesis, and sulfur within the Paaratte Formation (Figure 3). Furthermore, the microbial oxidation of sulfide, to recycle sulfur in its most reduced state to more oxidized forms, is preferentially facilitated by nitrate as the electron acceptor under anoxic conditions (Canfield and Thamdrup, 1994; Thamdrup et al., 1994; Habicht and Canfield, 1997; Bruchert and Pratt, 1999). Therefore, metagenomic analysis of sulfide:quinone oxireductase and the sulfur oxidation system (Poser et al., 2014), along with adenosine-5′-phosphosulfate reductase (*apr*A) (Meyer and Kuever, 2007) and dissimilatory sulfite reductase (*dsr*AB) genes (Geets et al., 2006) may provide further insights (Figure 3).

## Concluding remarks

Many subsurface environments targeted for CO_2_ storage have shown differences in microbial community composition. Thus, by using an analytical approach that considers potential syntrophic behaviors, this focused review on the geomicrobiology of geosequestration provides insights into more specialized biogeochemical processes—processes and relationships that would otherwise be omitted from solely taxonomical analyses—to reveal the complexity of what appears to be an extreme but simple environment (i.e., relatively low biomass, reduced conditions, high pressure and salinity). Co-occurrence profiling of the Paaratte Formation suggests the lack of an interdependency of the predominant OTUs with the majority of the biosphere, which implies a high degree of functional redundancy such that their removal may not affect the overall dynamics of the community. The review also highlights the need to consider anthropogenic activities in the subsurface as an engineered system in order to comprehend microbial community function and dynamics. Recent studies (e.g., Mitchell et al., 2008, 2009; Bordenave et al., 2012; Lavalleur and Colwell, 2013; Lau et al., 2014; Wilkins et al., 2014) are laying a foundation upon which we can start to build our understanding of the complexities of the subsurface biosphere. Indeed, future studies need to incorporate multi-omic approaches in order to unravel the interactions (the *interactome*; Baker, 2013) that connect subsurface biogeochemical processes. More importantly, the co-occurrence profiling approach described in this Focused Review can be a powerful *in silico* technique through which hypotheses are generated and specific subsurface populations tested *in vitro*, so as to start to address the gaps in our understanding of the complete geomicrobiological response to CO_2_ geosequestration.

### Conflict of interest statement

The authors declare that the research was conducted in the absence of any commercial or financial relationships that could be construed as a potential conflict of interest.
